# Exogenously Applied Proline Enhances Morph-Physiological Responses and Yield of Drought-Stressed Maize Plants Grown Under Different Irrigation Systems

**DOI:** 10.3389/fpls.2022.897027

**Published:** 2022-07-14

**Authors:** Abd El-Aty Ibrahim, Taia Abd El Mageed, Yasmin Abohamid, Hanan Abdallah, Mohamed El-Saadony, Synan AbuQamar, Khaled El-Tarabily, Nasr Abdou

**Affiliations:** ^1^Department of Soils and Water, Faculty of Agriculture, Fayoum University, Fayoum, Egypt; ^2^Department of Botany and Microbiology, Faculty of Science, Zagazig University, Zagazig, Egypt; ^3^Department of Agricultural Microbiology, Faculty of Agriculture, Zagazig University, Zagazig, Egypt; ^4^Department of Biology, College of Science, United Arab Emirates University, Al-Ain, United Arab Emirates; ^5^Khalifa Center for Genetic Engineering and Biotechnology, United Arab Emirates University, Al-Ain, United Arab Emirates; ^6^Harry Butler Institute, Murdoch University, Murdoch, WA, Australia

**Keywords:** deficit irrigation, irrigation system, maize, plant water status, proline, water productivity

## Abstract

The exogenous application of osmoprotectants [e.g., proline (Pro)] is an important approach for alleviating the adverse effects of abiotic stresses on plants. Field trials were conducted during the summers of 2017 and 2018 to determine the effects of deficit irrigation and exogenous application of Pro on the productivity, morph-physiological responses, and yield of maize grown under two irrigation systems [surface irrigation (SI) and drip irrigation (DI)]. Three deficit irrigation levels (I_100_, I_85_, and I_70_, representing 100, 85, and 70% of crop evapotranspiration, respectively) and two concentrations of Pro (Pro_1_ = 2 mM and Pro_2_ = 4 mM) were used in this study. The plants exposed to drought stress showed a significant reduction in plant height, dry matter, leaf area, chlorophyll content [soil plant analysis development (SPAD)], quantum efficiency of photosystem II [Fv/Fm, Fv/F0, and performance index (PI)], water status [membrane stability index (MSI) and relative water content (RWC)], and grain yield. The DI system increased crop growth and yield and reduced the irrigation water input by 30% compared with the SI system. The growth, water status, and yield of plants significantly decreased with an increase in the water stress levels under the SI system. Under the irrigation systems tested in this study, Pro_1_ and Pro_2_ increased plant height by 16 and 18%, RWC by 7 and 10%, MSI by 6 and 12%, PI by 6 and 19%, chlorophyll fluorescence by 7 and 11%, relative chlorophyll content by 9 and 14%, and grain yield by 10 and 14%, respectively, compared with Pro_0_ control treatment (no Pro). The interaction of Pro_2_ at I_100_ irrigation level in DI resulted in the highest grain yield (8.42 t ha^–1^). However, under the DI or SI system, exogenously applied Pro_2_ at I_85_ irrigation level may be effective in achieving higher water productivity and yield without exerting any harmful effects on the growth or yield of maize under limited water conditions. Our results demonstrated the importance of the application of Pro as a tolerance inducer of drought stress in maize.

## Introduction

Maize is one of the main cereal crops worldwide, followed by wheat, which is a staple food in Egypt (OECD/FAO, 2020). It is either consumed freshly or indirectly as corn oil, starch, fructose, glucose, and livestock feed (Ranum et al., 2014). Owing to its low inherent production and high demand of maize, Egypt has become the fourth largest importer of maize in the world (OECD/FAO, 2020). The total maize production in Egypt is approximately 6.4 million tons, and 85,000 hectares of cultivated land is estimated for its production. Its consumption is approximately 16.1 million tons with a self-sufficiency ratio of 42% (Ibrahim et al., 2007). Thus, to minimize the gap between maize production and consumption, it is important to manage irrigation water more efficiently and enhance domestic maize production by following non-traditional procedures, such as growing in cultivated areas or planting high-yield varieties (Ouda et al., 2017).

In arid and semi-arid regions, water shortage particularly affects food security (Misra, 2014; Mancosu et al., 2015). Approximately 70–80% of the available freshwater is required for agriculture (Food and Agriculture Organization [FAO], 2017). Egypt depends on limited water resources from the Nile River (55 billion m^3^ of water/year) (MWRI, 2014). Over 95% of irrigated lands in Egypt are managed using surface irrigation (SI) systems, with a low irrigation efficiency of 45–50% (Osman et al., 2016; Abdou et al., 2021; Ali et al., 2021). Therefore, rational water governance seeks to reduce water losses and enhance crop productivity to withstand high evaporative demand. Assuming severe water supply shortages, a deficit and highly efficient irrigation strategy of drip irrigation (DI) system is highly recommended over the SI system to increase the benefits per unit of water.

The application of irrigation water below the evapotranspiration (ET) demand is known as deficit irrigation, which optimizes economic output when there is limited water supply (Pereira et al., 2012; Chai et al., 2016). Plants under deficit irrigation systems receive less irrigation water than the actual amount of water required at plant growth stages and/or during the total crop cultivation period (Badal et al., 2013). Hence, plants are exposed to water stress to some extent under deficit irrigation systems (Wakchaure et al., 2018; Parkash and Singh, 2020). A decrease in the ET rate of plants exposed to water stress results in severe water stress symptoms, such as leaf rolling, diminishing leaf area, and stunted growth and yield of plants (Wang et al., 2014). Drought stress induces several physiological, biochemical, and photosynthetic changes by regulating stomatal closure or controlling CO_2_ flow into the mesophyll tissues (Yuan et al., 2016).

Approximately 20–25% of the maize cultivation areas are affected by drought globally (Shahrokhi et al., 2020). Sensitivity to drought stress can lead to dramatic fluctuations in maize yield, which is a common condition in Egypt (Gomaa et al., 2017). Maize exhibits distinct responses to water deficit depending on the developmental stage, crop tolerance level, and severity of the applied water stress treatments (Song et al., 2019; Cheng et al., 2021). The yield reduction due to water stress is primarily attributed to the disruption of physiobiochemical processes, inhibition of photosynthesis, and stunted plant growth and development (Song et al., 2019; Sharma et al., 2020; Cheng et al., 2021).

However, several studies have revealed that plants tend to accumulate various compatible solutes, such as soluble sugars and amino acids [e.g., proline (Pro)], as an adaptive tolerance strategy to increase salinity and/or drought tolerance (Giri, 2011; Semida et al., 2015; Arteaga et al., 2020). Pro is an essential amino acid that accumulates in various plant tissues, particularly in the leaves (Hayat et al., 2012). Proline accumulation plays an indispensable role in the regulation of cell osmosis (El Moukhtari et al., 2020). Additionally, under unfavorable conditions of water stress, Pro stabilizes membranes (Xia et al., 2020) and protects enzymes (Qamar et al., 2019; El-Nashaar et al., 2020), proteins, and macromolecules from denaturation (Agami et al., 2019). The effectiveness of exogenously applied Pro depends on the plant developmental stage and variety, Pro application rate, and the time of application (Semida et al., 2020). Excessive concentrations of free Pro can disrupt cell growth and protein functions. Hayat et al. (2007) observed that the foliar application of Pro (30 mM) negatively affects maize plants.

Using Pro as an antioxidant is an effective, unconventional, and inexpensive approach for mitigating the harmful effects of drought on maize production under different irrigation systems. Further studies are highly recommended to reduce the gap between water consumption and production of maize in arid and semi-arid regions. Therefore, this study aimed to elucidate the ameliorative effects of Pro at different nontoxic application rates on the morph-physiological and yield attributes of maize plants exposed to water stress. Furthermore, irrigation water requirements for maize plants grown under different irrigation systems and water scarcity conditions were determined.

## Materials and Methods

### Experimental Site

Field experiments were performed in the summers of 2017 and 2018 at the Agricultural Research Station in the Fayoum University, Fayoum Governorate, Egypt (latitude, 29°02′ and 29°35′ N and longitude, 30°23′ and 31°05′ E). The experimental treatments were arranged in a spilt-split plot in a completely randomized block design with three replications. The two irrigation systems, SI and DI, were set in the main plots. Three deficit irrigation levels (I_100_, I_85_, and I_70_ of ET) were employed in the subplots, and three concentrations of Pro (Pro_0_ = 0/control; Pro_1_ = 2 mM; and Pro_2_ = 4 mM) were used in the sub-sub plots.

### Initial Soil Characteristics of the Experimental Site

The soil in the field experiment was sandy loam; the water content at −0.33 and −15 bar pressure was retained by 20.52 and 8.87%, respectively. At a soil depth of 0.0–60 cm, the dry bulk density of the soil was 1.47 mg m^–3^, saturated hydraulic conductivity was 2.34 cm h^–1^, mean soil pest extract (ECe) was 3.75 dS m^–1^, and pH (soil:water suspension, 1:2.5) was 7.77. At the same soil depth, the mean organic matter and CaCO_3_ concentration were 13.8 and 87.8 g kg^–1^, respectively (Table 1).

**TABLE 1:** Some initial properties of the experimental soil samples.

Soil depth (cm)	Particle size distribution				ρb (Mg m^–3^)	Porosity %	Ks (cm h^–1^)	Soil moisture constants % at:			pH (1: 2.5 soil-water suspension)	ECe (dS m^–1^)	CaCO_3_ g kg^–1^	OM g kg^–1^
	Sand %	Silt %	Clay %	Texture class				FC	WP	AW				
0–20	73.40	11.30	15.30	S.L.	1.42	46.41	2.56	21.36	9.41	11.95	7.75	4.24	91.8	16.7
20–60	75.04	10.93	14.03	S.L.	1.52	42.86	2.12	19.68	8.32	11.36	7.79	3.25	83.9	10.9
Mean	74.23	11.11	14.66	S.L.	1.47	44.64	2.34	20.52	8.87	11.66	7.77	3.75	87.85	13.8

*SL, sandy loam; pb, bulk density; Ks, hydraulic conductivity; FC, field capacity; WP, wilting point; AW, available water; ECe, electrical conductivity; OM, organic matter.*

The initial physical and chemical characteristics of the soil at the experimental site were determined according to the methods proposed by Page et al. (1982) and Klute and Dirksen (1986).

### Experimental Treatments

#### Irrigation Systems

The SI and DI systems were implemented and installed at the experimental site as described in the following section.

##### SI System

Under the SI system, the quantity of irrigation water applied (IWA) for each plot was mainly controlled using a plastic pipe (spiles) with a diameter of 2 inches. For each experimental plot, one spile was constructed to direct the irrigation water. The quantity of IWA was estimated using the following equation (Israelsen and Hansen, 1962):


$$\text{Q}=\text{CA}\,\sqrt{2\,g\,\text{h}}\times 10^{-3}$$

where Q is the irrigation water discharge (L s^–1^), C is the discharge coefficient, A is the cross-sectional area of the spile (cm^2^), g is gravity acceleration (cm s^–2^), and h is the mean of the influential head of water (cm) above the pipe.

##### DI System

Under the DI system, irrigation water was provided through polyvinyl chloride pipes. A distance of 70 cm was maintained between the lateral lines, and emitters were spaced at 30 cm. An emitter discharge rate of 2 L h^–1^ was achieved with pumping at a pressure of 2 bar.

#### Water Stress Treatments

Three different water stress levels [I_100_, I_85_, and I_70_ (%) of ET_c_] were implemented. Maize plants were irrigated with the corresponding amounts of irrigation water, which were determined according to the daily reference crop ET as follows (Allen et al., 1998):


$${\text{ET}}_{\text{o}}={\text{E}}_{\text{pan}}\times {\text{K}}_{\text{pan}}$$

$${\text{ET}}_{\text{c}}={\text{ET}}_{\text{o}}\times {\text{K}}_{\text{c}}$$

where E_pan_ is the evaporation rate from Class A pan (mm day^–1^) and K_pan_ is the pan evaporation coefficient (0.85). ET_c_ is the sum of the water evaporation from the soil surface and transpiration (water loss) primarily from the plant leaves. ET_o_ is the reference ET. K_c_ is the crop coefficient; the K_c_ values at the initial, mid, and end stages were 0.70, 1.20, and 0.35, respectively. The irrigation water requirements (IWR) for each plot were determined using the following equation (Abd El-Wahed and Ali, 2013):


$$\text{IWR}=\frac{\text{A}\times \text{ETc}\times \text{Ii}\times {\text{K}}_{\text{r}}}{{\text{E}}_{\text{a}}\times 1000}$$

where IWR is the water requirement for irrigation (m^3^), A is the irrigated plot area (m^2^), ET_c_ is the crop ET (mm day^–1^), Ii is the interval between irrigations (days), Kr is the coverage coefficient, and E_a_ is the irrigation efficiency (%).

#### Antioxidant Applications

The antioxidant Pro was applied at three different concentrations : Pro_0_ (control) = 0, Pro_1_ = 2 mM, and Pro_2_ = 4 mM. It was applied three times as a foliar spray [30, 45, and 60 days after sowing (DAS)].

### Meteorological Parameters

**FIGURE 1:**
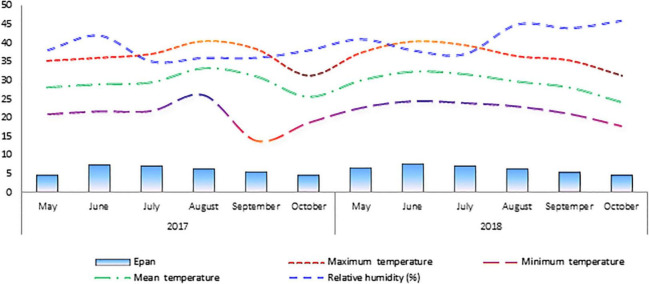
Monthly meteorological parameters in Fayoum Governorate in both seasons (2017 and 2018). The evaporation rate E_pan_ (mm day^−1^).

The monthly weather data for the summers of 2017 and 2018 were collected from the meteorological station of the Fayoum Governorate, Egypt (Figure 1). The climate in the experimental field was arid and characterized by low or no precipitation. During the period from May to September of each year, the maximum and minimum air temperatures were 40.4^°^C and 20.15^°^C, respectively, and the relative humidity fluctuated from 35 to 42%. The mean E_pan_ values during the cultivation period (May–September) were 6.48 and 6.50 mm day^–1^ for the first and second growing seasons, respectively.

### Agricultural Management Practices

Maize (*Zea mays*, hybrid 321) was cultivated on June 5 during the summers of both years (2017 and 2018) in hills that were 30 cm apart, with a distance of 70 cm between the lateral lines. The experimental plots were fertilized using N:P:K (200:100:75; kg ha^–1^). Total superphosphate (15.5% P_2_O_5_) along with potassium sulfate (48% K_2_O) was applied before cultivation. Nitrogen fertilizer was applied using two equivalent doses, with the first and second doses applied at the first and second irrigation events, respectively.

All other agricultural operations required for the growth and development of maize plants were performed similarly in all plots according to the recommendations of the Egyptian Ministry of Agriculture. Maize plants were harvested 120 DAS.

### Physiological Maize Parameters

Ten plant samples (70 DAS) were randomly selected from each plot to measure their physiological responses to the treatments applied.

### Relative Water Content

The leaf samples for relative water content (RWC) measurement were randomly collected in the morning (8:00 a.m.–9:00 a.m.). RWC (%) was determined according to the following equation (Hayat et al., 2007):


$$\mathrm{RWC}\left(\%\right)=\frac{\text{FW}-\text{DW}}{\text{TW}-\text{DW}}\times 100$$

where FW is the fresh weight measured within 2 h after the excision of leaves; TW is the turgid weight computed by soaking the leaves in distilled water and leaving them at room temperature for 16–18 h, followed by rapid and careful dry-blotting on tissue paper. The small leaf pieces were later oven-dried at 70°C for 48 h to assess the dry weight (DW).

### Leaf Membrane Stability Index

MSI (%) was measured using the method described by Premachandra et al. (1990). Small leaf strips (0.2 g) of equal size were prepared and placed in two sets of test tubes, each containing 10 mL of distilled water. The test tubes of the first set were incubated in a water bath at 40^°^C for 30 min, and ECe was subsequently estimated (C_1_), whereas those of the second set were incubated in a boiling water bath at 100^°^C for 15 min, followed by ECe measurement (C_2_). MSI = $[1-\frac{\mathrm{C1}}{\mathrm{C2}}\text{]}\times 100$

### Relative Chlorophyll Content Values

The relative chlorophyll content SPAD was determined using SPAD 502 (Konicaminolta. Inc., Tokyo), as described by Maxwell and Johnson (2000).

### Chlorophyll Fluorescence

Chlorophyll fluorescence (Fv/Fm) was determined using a portable fluorometer (Spoustová et al., 2013).

### Performance Index

The performance index (PI) of photosynthesis was determined according to the method proposed by Clark et al. (2000).

During harvesting (120 DAS), ten plants from each plot were collected to determine their height (cm), stem diameter (cm), leaf number plant^−1^, root weight, cob weight (g), 100-grain weight (g), grain yield (t ha^–1^), and biomass yield (t ha^–1^).

### Water Productivity

Water productivity (WP) is expressed as the grain yield (kg) per IWA (m^3^). The values were used to evaluate the variation in different treatments for producing the maximum yield from the water unit consumed by the maize plants. The WP values were calculated (Jensen, 1983) as per the following equation:


$$\text{WP}=\frac{\mathrm{Grain}\,\mathrm{yield}\,\left(\mathrm{kg}\,{\text{ha}}^{-1}\right)}{\mathrm{Irrigation}\,\mathrm{water}\,\mathrm{applied}\,\left({\text{m}}^{3}\,{\text{ha}}^{-1}\right)}$$

### Statistical Analysis

The study was designed in a completely randomized block design (spilt-split plot) with three replications, and the obtained data were statistically analyzed according to the procedures outlined by Gomez and Gomez (1984) using the GenStat statistical package, 12th edition (VSN International Ltd., Oxford, United Kingdom).

## Results

### Morphological Characteristics of Maize Plants

Table 2 indicated that all maize growth parameters, i.e., plant height (cm), stem diameter (cm), leaf number plant^−1^, and root weight (g), were significantly affected by the applied water stress levels, exogenous Pro treatments, and their interactions under different irrigation systems. Plants under the DI system showed a considerable increase in the plant height (17.82%), stem diameter (17.86%), and leaf number plant^−1^ (18.77%) compared with those under the SI system. An exception was observed for the root weight in which the highest value (54.85 g plant^−1^; mean value of both seasons) was observed under the SI system. Additionally, fully irrigated maize plants (I_100_) exhibited higher growth rates than drought-stressed maize plants. Compared with the control (I_100_), the mean values (during the summers of both years) of plant height, stem diameter, leaf number plant^−1^, and root weight decreased by 10.51, 9.61, 3.01, and 11.14%, respectively, at a moderate stress level (I_85_). These values decreased by 17.38, 22.55, 10.94, and 20.60%, respectively, under a severe stress level (I_70_).

**TABLE 2:** Effect of water stress treatments and proline application rates on maize growth traits under different irrigation systems.

Source of variation	Plant height (cm)		Stem diameter (cm)		Number of leaves plant^–1^		Roots weight (g)	
	S_I_	S_II_	S_I_	S_II_	S_I_	S_II_	S_I_	S_II_
IS	**	**	**	**	**	**	**	**
SI	169.51b	171.63b	1.95b	1.97b	12.83b	13.82b	54.4a	55.31a
DI	199.81a	202.13a	2.29a	2.33a	15.25a	16.4a	49.07b	48.99b
I	**	**	**	**	**	**	**	**
I_100_(control)	205.22a	204.41a	2.38a	2.41a	14.63a	15.96a	58.56a	57.79a
I_85_	181.54b	185.01b	2.14b	2.19b	14.23a	15.42a	51.27b	52.11b
I_70_	167.21c	171.21c	1.84c	1.87c	13.27b	13.95b	45.39c	46.98c
Pro	*	**	**	**	**	**	**	**
Pro_0_ (control)	164.45c	166.53c	1.77c	1.79b	12.54c	13.75b	42.67c	43.03c
Pro_1_	189.98b	192.34b	2.21b	2.28a	14.41b	15.59a	54.71b	54.82b
Pro_2_	199.54a	201.78a	2.38a	2.40a	15.18a	15.99a	57.83a	58.61a
IS × I	*	**	*	**	*	**	*	*
IS × Pro	**	**	**	*	*	*	**	NS
I × Pro	*	*	*	*	*	**	*	*
IS × I × Pro	**	NS	NS	NS	NS	NS	NS	NS

* and ** refer to the significant difference at p ≤ 0.05 and p ≤ 0.01, respectively.

*S_I_, the first season; S_II_, the second season; IS, irrigation system; SI, surface irrigation; DI, drip irrigation; I, irrigation regime; Pro, proline treatment (Pro_0_ = 0, control; Pro_1_ = 2 mM; and Pro_2_ = 4 mM); NS, not significantly different. In each column the different letters attached to the mean values indicates significant difference according to Duncan’s multiple range test.*

The exogenously applied Pro ameliorated the adverse effects of drought stress on maize plants. The maximum plant height, stem diameter, leaf number plant^−1^, and root weight were 199.54 cm, 2.39 cm, 15.58, and 58.22 g, respectively, for plants treated with Pro_2_ during the summers of both years. After increasing the level of the exogenously applied Pro, the plant height, stem diameter, leaf number plant^−1^, and root weight under Pro_1_ treatment increased by 15.51, 26.12, 14.15, and 27.81%, whereas those under Pro_2_ treatment increased by 21.25, 34.27, 18.67, and 35.87%, respectively, compared with the control (Pro_0_).

### Plant Water Status (RWC and MSI)

As shown in Table 3, the two indicators of plant water status, RWC (%) and MSI, showed a significant response to water stress and Pro treatments under the two irrigation systems during the summers of both years, whereas the combined effect of these treatments (IS × I × P) on the aforementioned indicators was not significant. The results showed that maize plants under the DI system exhibited better physiological responses than those under the SI system. Additionally, water stress considerably reduced RWC by 10.51 and 17.38% in maize plants under moderate (I_85_) and severe (I_70_) stress levels, respectively, compared with those under no stress (controls; I_100_). The exogenous application of Pro mitigated the drought-induced inhibitory effects on RWC. Compared with the control (Pro_0_), the mean values of RWC increased by 15.51 and 21.25% with increased concentrations of Pro_1_ and Pro_2_, respectively. Furthermore, the highest mean MSI (67.98%) was observed in adequately irrigated plants (100% ET_c_), and the lowest mean MSI (59.69%) was observed in plants exposed to high water deficit levels (I_70_). Cell membrane integrity is highly susceptible to drought stress. Water stress leads to the disruption of cell membrane stability, thus increasing electrolyte leakage and decreasing membrane integrity. The mean MSI increased by 6.88 and 13.25% for Pro_1_- and Pro_2_-treated plants, respectively, compared with that for non-Pro-treated plants during the summers of both years.

**TABLE 3:** Effect of water stress and proline application rates on relative water content (RWC), membrane stability index (MSI), chlorophyll fluorescence (Fv/Fm), performance index (PI), and relative chlorophyll content (SPAD) values under different irrigation systems.

Source of variation	RWC %		MSI %		Fv/Fm		PI		SPAD	
	S_I_	S_II_	S_I_	S_II_	S_I_	S_II_	S_I_	S_II_	S_I_	S_II_
IS	**	**	**	**	* *	**	**	* *	**	**
SI	70.82b	71.76b	59.4b	58.96b	0.72b	0.72b	2.10b	2.12b	34.32b	34.39b
DI	76.67a	77.51a	69.51a	68.76a	0.77a	0.78a	2.46a	2.48a	41.22a	42.1a
I	**	**	**	**	**	**	**	**	**	**
I_100_(control)	78.94a	80.68a	68.12a	67.85a	0.78a	0.79a	2.68a	2.71a	41.39a	43.41a
I_85_	74.11b	73.87b	64.93b	64.69b	0.75b	0.76a	2.22b	2.24b	38.12b	38.51b
I_70_	68.19c	69.38c	60.34c	59.05c	0.71c	0.71b	1.94c	1.94c	33.79c	32.84c
Pro	**	*	*	**	**	**	**	*	**	**
Pro_0_ (control)	70.06c	69.98c	59.94c	60.31c	0.70c	0.71c	2.09c	2.11c	35.21c	35.15c
Pro_1_	74.48b	75.70b	64.94b	63.58b	0.75b	0.76b	2.24b	2.23b	38.25a	38.37b
Pro_2_	76.71a	78.23a	68.5a	67.68a	0.79a	0.79a	2.52a	2.55a	39.86a	41.24a
IS × I	**	NS	NS	NS	NS	NS	NS	NS	NS	NS
IS × Pro	*	NS	NS	NS	NS	NS	NS	NS	NS	NS
I × Pro	**	NS	**	NS	NS	*	**	*	NS	NS
IS × I × Pro	*	NS	*	NS	NS	NS	NS	NS	NS	NS

* and ** refer to the significant difference at p ≤ 0.05 and p ≤ 0.01, respectively.

*S_I_, first season; S_II_, second season; IS, irrigation system; SI, surface irrigation; DI, drip irrigation; I, irrigation regime; Pro, proline treatment (Pro_0_ = 0, control; Pro_1_ = 2 mM; and Pro_2_ = 4 mM); NS, not significantly different. In each column the different letters attached to the mean values indicates significant difference according to Duncan’s multiple range test.*

### Fv/Fm, PI, and Soil Plant Analysis Development

The data presented in Table 3 revealed that maize plants showed significant differences in Fv/Fm, PI, and SPAD in response to water stress and Pro foliar treatments under the SI and DI systems during the summers of both years; however, the effect of their interaction was not significant. The values for these traits increased by 7.64, 17.06, and 21.26% for Fv/Fm, PI, and SPAD, respectively, under the DI system compared with the SI system. Water stress adversely affects photosynthesis. The Fv/Fm, PI, and SPAD values showed a consistent decrease with increasing water deficit. Compared with sufficiently irrigated plants (I_100_), the Fv/Fm, PI, and SPAD values decreased by 3.82, 17.25, and 9.59%, respectively, in plants grown under the moderate-deficit irrigation level (I_85_) and by 9.55, 28.01, and 21.36%, respectively, in those under the high-deficit irrigation level (I_70_). Furthermore, this study indicated that the harmful effects of drought stress on photosynthesis can be alleviated in maize through foliar Pro application. The exogenous application of Pro (Pro_2_ concentration) on maize plants exposed to water stress increased the Fv/Fm, PI, and SPAD values by 12.06, 20.71, and 15.27%, respectively, compared with the control (Pro_0_).

### Yield and Yield Components

Table 4 showed that the yield and yield components of maize, such as cob weight (g), 100-grain weight (g), grain yield (t ha^–1^), and biomass yield (t ha^–1^), differed significantly in response to water stress and Pro applications under the SI and DI systems during the summers of both years. The effects of interaction were not significant between (IS × I) and (IS × P) for the 100-grain weight parameter. Except of grain yield, the interaction effect (IS × IR × P) on all abovementioned traits was not significant (*P* > 0.05). Maize plants exposed to water stress had a lower yield than fully irrigated plants. Compared with the full irrigation level (I_100_), the cob weight (g), 100-grain weight (g), grain yield (t ha^–1^), and biomass yield (t ha^–1^) of maize plants decreased by 4.33, 4.45, 6.36, and 9.24%, respectively, at the moderate-deficit irrigation level (I_85_) and by 7.49, 14.90, 25.44, and 17.97% at the high-deficit irrigation level (I_70_). The exogenously applied Pro increased the yield of maize plants under deficit irrigation; the cob weight, 100-grain weight, grain yield, and biomass yield increased (as mean values of both seasons) by 11.70, 21.07, 10.93, and 15.44%, respectively, at moderate Pro (Pro_1_) application rates and by 15.61, 27.89, 15.99, and 23.05%, respectively, at high Pro (Pro_2_) application rates, compared with the control (Pro_0_).

**TABLE 4:** Effect of water stress and proline application rates on yield and yield components of maize crop under different irrigation systems.

Source of variation	Cob weight (g)		100 Grains weight (g)		Grains yield (t ha^–1^)		Biomass yield (t ha^–1^)	
	S_I_	S_II_	S_I_	S_II_	S_I_	S_II_	S_I_	S_II_
IS	**	**	**	**	**	**	**	**
SI	117.90b	119.72b	29.73b	30.18b	6.07b	6.08b	31.01b	32.09b
DI	138.07a	138.65a	34.95a	35.87a	7.27a	7.30a	37.14a	38.13a
I	**	**	**	**	**	**	*	*
I_100_(control)	133.35a	134.37a	34.73a	35.15a	7.47a	7.47a	37.55a	38.54a
I_85_	127.36b	128.78b	32.94b	33.83b	6.98b	7.01a	34.02b	35.04b
I_70_	123.25c	124.41c	29.36c	30.11c	5.56c	5.58b	30.66c	31.76c
Pro	**	**	**	**	**	**	**	**
Pro_0_ (control)	117.47c	118.25c	27.97c	28.23c	6.11c	6.15c	30.13c	31.19c
Pro_1_	131.16b	132.15b	33.95b	34.09b	6.80b	6.80b	34.93b	35.85b
Pro_2_	135.33a	137.18a	35.11a	36.77a	7.10a	7.12a	37.17a	38.28a
IS × I	**	**	NS	NS	**	*	**	**
IS × Pro	*	**	NS	NS	**	**	*	**
I × Pro	**	**	*	**	**	**	*	*
IS × I × Pro	*	NS	NS	NS	*	*	NS	NS

* and ** refer to the significant difference at p ≤ 0.05 and p ≤ 0.01, respectively.

*S_I_, first season; S_II_, second season; IS, irrigation system; SI, surface irrigation; DI, drip irrigation; I, irrigation regime; Pro, proline treatment (Pro_0_ = 0, control; Pro_1_ = 2 mM; and Pro_2_ = 4 mM); NS, not significantly different. In each column the different letters attached to the mean values indicates significant difference according to Duncan’s multiple range test.*

### Irrigation Water Applied

Table 5 showed that IWA varied according to the designed irrigation regime and system. The highest values of irrigation water inputs were observed under the SI system at the full irrigation level. The need for irrigation gradually decreased as the water deficit level increased in the DI system because its high-efficiency design saved 29.41% of IWA compared with the SI system.

**TABLE 5:** Irrigation water applied (m^3^ ha^–1^) for different irrigation levels and irrigation systems.

Irrigation system	Irrigation treatment					
	I_100_		I_85_		I_70_	
	S_I_	S_II_	S_I_	S_II_	S_I_	S_II_
IS	7,754	7,786	6,591	6,618	5,427	5,450
D	5,473	5,496	4,652	4,672	3,831	3,847

*S_I_, first season; S_II_, second season; I, irrigation; IS, irrigation system; D, drought.*

### Water Productivity

**FIGURE 2:**
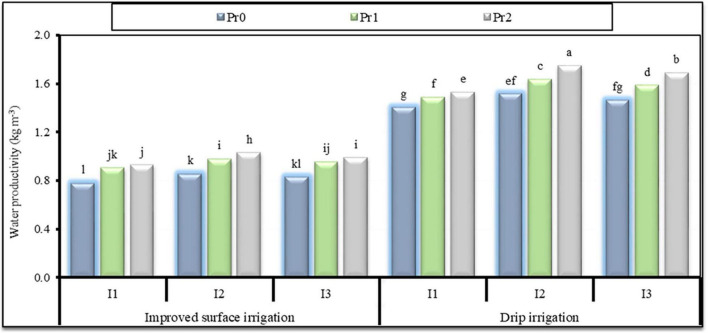
Effect of water stress and proline (Pr) application rates (Pr_0_ = 0, control; Pr_1_ = 2 mM; and Pr_2_ = 4 mM), on water productivity (as mean values of the two seasons) under different irrigation systems. Different letters on the bars refer to significant differences among means based on Fisher’s least significant difference test at the *p* < 0.05 level.

As shown in Figure 2, the highest WP (1.80 kg m^–3^) was observed at I_85_ with a high Pro application rate (Pro_2_) under the DI system. In contrast, the lowest (0.79 kg m^–3^) WP was observed at I_100_ with a low Pro application rate (Pro_1_) under the SI system. For each irrigation level and Pro application treatment, DI considerably enhanced WP compared with SI. DI delivers water near each plant, leading to high water-use efficiency. Among the water stress treatments, the irrigation regime of I_85_, in which the reduction in grain yield was lower than the amount of irrigation water saved, showed the highest WP-value compared with other irrigation regimes.

### Effect of Irrigation Systems and Irrigation Regimes on Salt Distribution Pattern

**FIGURE 3:**
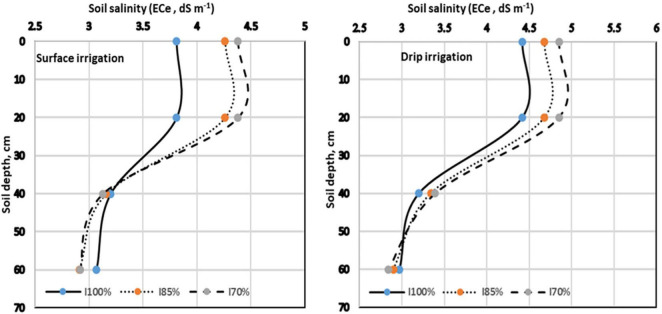
Effect of irrigation systems and irrigation regimes on salt distribution pattern. ECe, soil electrical conductivity.

Figure 3 showed that SI, particularly when coupled with high levels of irrigation water (I_100_), enhanced the downward movement or migration of soluble salts, thus reducing the concentrations of salt accumulated in the lower soil layers by 10.14% compared with those accumulated on the soil surface (0–20 cm). In contrast, DI with low irrigation input (I_70_) promoted the upward movement of salts, thus increasing soil salinity in the same layer (0–20 cm) by 14.38% compared with the initial soil salinity.

## Discussion

Water stress adversely affects the growth and development of plants, particularly when the stress conditions remain constant. When plants are exposed to stress, they accumulate an array of metabolites, such as amino acids. Pro plays a highly beneficial role in plants exposed to various stress conditions. Besides acting as an excellent osmolyte, Pro has three fundamental biological roles in stress response, i.e., acting as a metal chelator, antioxidative defense molecule, and signaling molecule. Some reports; however, have indicated the toxic effects of Pro when applied exogenously at high concentrations. In this study, we aimed to (i) investigate the causes underlying the reduction in the growth and yield of maize plants induced by water stress and (ii) determine the crucial role of Pro in mitigating these negative effects at different nontoxic application rates of Pro to improve WP under water scarce conditions.

Our results demonstrated significant differences in the vegetative growth of maize plants in response to the two irrigation systems (Table 2). The DI system was relatively superior in the maize growth parameters, except for root weight, compared with the SI system. Similar to previous reports (Ghamarnia et al., 2013; Mahgoub et al., 2017), the observed increase in the root weight of plants under the SI system could be a result of the downward movement of irrigation water due to gravity, which promotes the penetration of roots through the soil profile to greater depths to extract water from the deeper layers. Maize plants exposed to water stress showed growth retardation. The inhibition of maize growth due to water stress was evident because of the decrease in the length, volume, and water potential of the root system, which disrupted the water extraction by the roots. This results in decreased cell division and elongation, leading to stunted plant growth. These results were consistent with those of previous studies (El-Samnoudi et al., 2019; Song et al., 2019; Sah et al., 2020; Cheng et al., 2021). However, the exogenous application of Pro not only provided osmoprotection but also enhanced the growth of the plants. The beneficial effect of Pro in enhancing maize growth characteristics under low irrigation levels might be attributed to its role in osmoregulation, and maintenance of the tertiary structure of proteins and enzymes, to help growing plants tolerate drought stress. These results were similar to those of the previous reports (Abdelaal et al., 2018, 2020; Qamar et al., 2019; El-Nashaar et al., 2020).

Additionally, drought adversely affected the plant water retention (RWC and MSI) and photosynthesis (Table 3). The decrease in water uptake under deficit irrigation regimes was associated with a decrease in leaf water potential. Drought alters the plant water status and stomatal functioning and inhibits chlorophyll biosynthesis, thus reducing the photosynthetic rates. Dehydration in the plant cell protoplasm could be considered as an effect of drought on the RWC of maize leaves. Similar observations were reported in a previous study (Xia et al., 2020). The foliar application of Pro enhanced the plant water status. This was evident by the reduction in water efflux under drought stress, which helped the cells maintain their cell turgor or osmotic balance. These results are consistent with those of another study conducted by Al-Khazrji et al. (2020). It is suggested that the exogenous application of Pro enhances membrane stability, thus preventing electrolyte leakage, as previously documented by Hayat et al. (2012).

Water stress further decreased the values of the physiological parameters in maize seedlings. This was evident by the disruption of plant photosynthetic potential, possibly due to the stomatal closure and/or metabolic destruction, such as the impairment of photosystem 1 (PSI) and photosystem II (PSII), which are chlorophyll-binding protein complexes (Ibrahim et al., 2019; Zheng et al., 2022). Furthermore, water stress increases the generation of reactive oxygen species (ROS), resulting in oxidative damage to plants and degradation of chlorophyll pigments (Hasanuzzaman et al., 2020; Mansoor et al., 2022). Alternatively, Pro may be responsible for scavenging ROS and other free radicals (Kaul et al., 2008). It may also protect plants from stress injuries by stabilizing membranes and proteins, allowing the transport of mitochondrial electrons, enhancing antioxidant enzyme activity, thus increasing stomatal conductance and facilitating higher CO_2_ diffusion through leaves. This promotes higher photosynthetic capacity (Tùmová et al., 2018; Agami et al., 2019; Altuntaş et al., 2020; Semida et al., 2020; Tariq et al., 2021; Abdou et al., 2022). Hence, the foliar application of Pro can enhance the photosynthetic rate of maize under drought stress.

The decreased water uptake from the soil might be responsible for the detrimental effects of water stress on grain and biomass yields of maize (Table 4), resulting in the abovementioned inhibition in plant growth and development, disruption of photosynthetic pigments, and deficits in plant water content. Accordingly, a significant decrease in maize yield under water stress conditions was observed. The results are consistent with those of previous studies (Vazirimehr et al., 2014; Sah et al., 2020; Shah et al., 2020).

The present study demonstrated that the increase in the yield and yield components achieved by the application of Pro was higher than the reduction in the yield caused by drought stress, confirming that Pro treatment successfully compensated for the adverse effects of drought stress on the growth and yield of maize. Furthermore, the yield and yield components of maize under the DI system were higher than those under the SI system (Qamar et al., 2019; Abdelaal et al., 2020; El-Nashaar et al., 2020; Li et al., 2021), and WP increased consistently with increasing concentrations of exogenously applied Pro (Table 5). These results are consistent with those of the previous studies by Semida et al. (2015) and Semida et al. (2020) who found that pressurized irrigation systems via subsurface drip and/or surface drip increase the water-use efficiency and grain yield of maize compared with the SI system.

Notably, the monitoring of the salt distribution pattern in the soil profile (depth, 0–60 cm) based on different applied irrigation systems and treatments showed that the salt distribution pattern was different after plant harvest in the second season compared with the determined initial ECe values before maize planting. The results were consistent with those of the studies by Semida et al. (2015) and Ali et al. (2016) who reported that the highest salinity was observed in the deeper soil layers depending on the wetting front under the SI system. The lowest salinity was observed on the surface; however, under DI, salt accumulation increased on the soil surface.

## Conclusion

With increasing water scarcity, well-designed deficit DI regimes and the exogenously applied antioxidant Pro can optimize the maize production and WP when the available water is insufficient to provide full irrigation. The DI system efficiently saved irrigation water input by 30% compared with the SI system. Among the tested irrigation levels, the full level (I_100_) resulted in the highest grain yield. The moderate-deficit irrigation level (I_85_) showed the maximum WP. The growth, physiological aspects, and grain yield of maize increased significantly after the foliar application of Pro to maize plants exposed to water stress. Therefore, using exogenous Pro at 4 mM for maize plants under the DI system and irrigating with 85% of their ET demand may be a promising agro-management strategy for improving the yield and WP of maize crops grown in arid and semi-aired regions, especially when water scarcity constraints the sustainability of maize production.

## Data Availability Statement

The original contributions presented in this study are included in the article, further inquiries can be directed to the corresponding author/s.

## Author Contributions

AE-AI, TA, YA, SA, KE-T, and NA: conceptualization. AE-AI, TA, YA, and NA: methodology. AE-AI, TA, SA, KE-T, and NA: formal analysis. TA, SA, KE-T, and NA: investigation and writing—original draft preparation. AE-AI, TA, YA, ME-S, HA, and NA: data curation. TA, ME-S, SA, KE-T, and NA: writing—review and editing. TA and HA: visualization. SA and KE-T: funding acquisition. All authors have read and agreed to the published version of the manuscript.

## Conflict of Interest

The authors declare that the research was conducted in the absence of any commercial or financial relationships that could be construed as a potential conflict of interest.

## Publisher’s Note

All claims expressed in this article are solely those of the authors and do not necessarily represent those of their affiliated organizations, or those of the publisher, the editors and the reviewers. Any product that may be evaluated in this article, or claim that may be made by its manufacturer, is not guaranteed or endorsed by the publisher.
